# Increasing rates of laparoscopic gastrointestinal surgery and decreasing rates of surgical site infections: an observational study in Japan from 2012–2017

**DOI:** 10.1186/s12893-021-01373-2

**Published:** 2021-10-20

**Authors:** Tomoko Takahashi, Hiroshi Nishiura

**Affiliations:** 1 Graduate School of Medicine, Hokkaido University, Kita 15 Jo Nishi 7 Chome, Kita-ku, Sapporo-shi, Hokkaido, 060-8638 Japan; 2 Infection Control Team, Sapporo Kousei Hospital, Kita3-Higashi8, Chuo-Ku, Sapporo, Hokkaido 060-0033 Japan; 3 Kyoto University School of Public Health, Yoshida-konoecho, Sakyoku, Kyoto, 606-8501 Japan

**Keywords:** Surgical site infection, Gastrointestinal surgery, Laparoscopic surgery, Surveillance, Epidemiology, Infection control

## Abstract

**Background:**

Surgical site infections (SSI) are the most common healthcare-associated infection, and gastrointestinal surgery is frequently followed by a high incidence of SSI. Epidemiological analysis of the temporal trends in SSI following gastrointestinal surgery has yet to be performed in Japan. Our purpose was to descriptively investigate these trends.

**Methods:**

Extracting national surveillance data from the Japan Nosocomial Infections Surveillance (JANIS) system, we analyzed the frequency of SSI events following gastrointestinal surgery, which consisted of seven surgical procedures, from 2012–2017. We calculated the standardized infection ratio to compute risk-adjusted SSI frequency, and used the trend test to detect time-dependent changes.

**Results:**

The frequency of SSI events, except for those associated with surgery of the upper gastrointestinal tract, revealed a decreasing time-dependent trend. The use of laparoscopic surgery increased dramatically over time (p < 0.01 for the six applicable surgical sites), while the frequency of SSI events during laparoscopic surgery remained unchanged.

**Conclusions:**

The increasing use of laparoscopic surgery was identified, and the observation coincided with the decreasing trend in SSI, especially in lower gastrointestinal tract surgery. If the relationship is causal, the overall SSI incidence among previously healthy individuals is expected to decrease, because the use of laparoscopic surgery has large growth potential in Japan.

**Supplementary Information:**

The online version contains supplementary material available at 10.1186/s12893-021-01373-2.

## Background

Surgical site infection (SSI) is one of the most common healthcare-associated infections, and the leading cause of longer hospital stay, higher mortality, and substantial medical costs [1–9]. Among all SSI events, the incidence of SSI is generally very high with gastrointestinal surgery, and especially so for colonic and rectal surgery, in several countries, including Japan [10, 11, 11, 12]. The incidence of SSI following gastrointestinal surgery in 2017 ranged from 0.6 to 24.9% in Japan, with the variation depending on the surgical site [10]. Understanding the detailed mechanisms of SSI following gastrointestinal surgery and considering possible countermeasures is of critical importance.

Several epidemiological studies have been performed both in Japan and in other countries [11, 11], exploring the possible predictive factors for incisional SSI or postoperative adverse events. Patient-related risk factors for SSI following gastrointestinal survey have been investigated in Japan, and identified factors in published studies were intraoperative blood transfusion, diabetes, and the use of steroids [13]. Risk factors other than patient-related factors have also been analyzed, namely, ileostomy or colostomy placement, emergency operation, and multiple colonic procedures [14]. Despite these previous studies [11, 11–15], no epidemiological studies have been performed in Japan to investigate the time trends in SSI incidence (or alternatively, trends in the standardized infection ratio or relative risk of specific risk factors) following gastrointestinal surgery, or to explore the mechanisms underlying the trends, as have been performed elsewhere [16].

The incidence of SSI associated with gastrointestinal surgery, inclusive of esophageal, stomach, small intestinal, colonic, and rectal surgery, has declined over time. However, depending on the surgical technique or procedures, the extent of the decrease varies, and the variability depends on the surgical site [17]. An international cohort study reported a consistent and substantial decline in SSI incidence for colorectal surgery [15]. Understanding the time trends for SSI incidence may help determine important features or risk factors regulating the risk of SSI for specific surgical sites, and may be critical in evaluating past countermeasures and considering possible future actions.

The purpose of the present study was to descriptively investigate the time-dependent characteristics of SSI incidence following gastrointestinal surgery in Japan. Because numerous risk factors have been investigated using individual-based datasets and addressing potential confounders [13, 14], the present study focused on the trend analysis, limited to adjusting for confounders only when calculating the standardized risk of infection. Using the trend analysis techniques, we examined the nosocomial infection surveillance data.

## Methods

### Surveillance data

This study used data from a healthcare-associated infection surveillance system, which collects, analyzes, and interprets data on outbreaks of healthcare-associated infections. The system is based on epidemiological principles, and results data are shared with those who can improve the results [18]. Japan has a nationwide nosocomial infection surveillance project, the Japan Nosocomial Infections Surveillance (JANIS) system, which is continuously managed by the Ministry of Health, Labour and Welfare [19]. Beginning in 2000 with the clinical laboratory division, antimicrobial-resistant bacterial infection division, and intensive care unit division, data were submitted voluntarily from medical institutions with ≥ 200 hospital beds. Since 2002, two more divisions, namely, the surgical site infection and neonatal intensive care unit divisions, have been added, and JANIS system renewal began in 2007 to allow public dissemination of the surveillance information online [19]. As an audit system, JANIS contacts each medical institute when reported datasets are in question. Moreover, the board meeting regularly reviews items for data collection and the results of statistical analyses, supported by expert members of the board. Furthermore, due to the nature of open datasets, any users can point out potential issues in the database [19].

Our study subject, the SSI division, follows the design of the SSI surveillance of the United States Centers for Disease Control and Prevention, which began in the 1980s. The JANIS SSI division has been fully operational since 2007. As of January 2019, 877 Japanese medical facilities have been registered in JANIS [19]. The present study used data from 2012–2017 because the data were electronically managed with a consistent format during this period. Openly accessible datasets for the following seven surgical techniques: esophagectomy, gastrectomy, total gastrectomy, distal gastrectomy, small bowel surgery, colon surgery, and rectal survey, were retrieved for analysis. In this context, total gastrectomy represents removing the entire stomach (i.e., total gastric resection), and distal gastrectomy is defined as pyloric-side gastrectomy with Billroth-I and Billroth -II reconstruction. Gastrectomy is defined as gastric incision and resection, excluding pyloric gastrectomy and total gastrectomy, and this definition does not include vagus nerve resection and cardioplasty. Esophagectomy involves both thoracoscopy and laparoscopy (but the SSI data were not classified according to thoracoscopy and laparoscopy), and the following descriptions of laparoscopy for esophagectomy actually refer to the use of either laparoscopy or thoracoscopy. In each year, the frequency of SSI events per operation was recorded according to the type of surgery (i.e., open or laparoscopic/thoracoscopic surgery), stoma construction (e.g., ileostomy or colostomy), elective or emergency operation, and sex (male or female). Additionally, partial datasets for wound classification, operation duration, and the American Society of Anesthesiologists score (representing patient status severity) were available for laparoscopic surgery and stoma datasets.

SSI was classified into three types, namely superficial incisional SSI, deep incisional SSI, and organ/space SSI, depending on the location of the SSI. One of the criteria for SSI is infection occurring within 30 days after non-implant surgery and within 1 year after implant surgery. SSI does not necessarily occur during the hospitalization period. Even after discharge, infection was judged as an SSI if it met the definition of SSI. Further details of the SSI definition are standardized by JANIS, which refers to the definitions by the United States Center for Disease Control and Prevention. To adjust for important confounders, SSI risk was stratified by known risk factors, namely operation time, patients’ background characteristics, and the type of surgical incision [19].

### Epidemiological risk

Our study outcome was SSI events. To understand the time-dependent trends in SSI, we explored SSI frequency by surgical technique (i.e., by operation site) and also examined the SSI frequency per medical institute, both calculated as the mean of all reporting institutes. Because the frequency of laparoscopic surgery for any gastrointestinal tract surgery increased dramatically in Japan during the study period, we stratified SSI frequency by the type of surgery. To quantify the contribution of laparoscopic surgery to SSI events, we calculated the relative risk of SSI, comparing laparoscopic against open surgery. Additionally, to correct for patient status severity using an indirect standardization method, we also calculated the standardized infection ratio (SIR) using the 2013 data as reference data, and applying the patient severity correction method to the SSI data. Subsequently, the relative risk and SIR of an SSI event were also computed by (i) the presence of a stoma (vs the absence of a stoma), (ii) emergency operation (vs elective operation), and (iii) sex (male vs female) over the course of the observation period.

With *R* as the risk of SSI events in a standard population; *i*, the identity of a stratum; *n*, the total number of strata; and *P*, the total number of surgical operations, for a stratum *i*, the total number of SSI events within the stratum is calculated as *R*_i_*P*_i_, and thus, the expected total number of SSI events across different *i* is indicated by the sum of *R*_i_*P*_i_ over *i*. Accordingly, the ratio of the total number of actual SSI events to the expected total number of SSI events is described by:

1 $$SIR=\frac{O}{\sum_{\mathrm{i}=1}^{\mathrm{n}}{R}_{i}{P}_{i}}.$$

Regarding the strata *i*, we accounted for reported scores for (i) wound classification (e.g., if the surgical wound was contaminated), (ii) operation duration, and (iii) preoperative general patient status as judged by the anesthesiologist. The denominator represents the number of SSI events that would have occurred if the year was 2013, and the numerator is the actual number of SSI events in a given year. The expected number of patients in the denominator estimates the expected number of SSI events related only to patient factors, and the number of SSI events in the numerator highlights patients’ factors and additional factors such as those associated with the medical facility [20]. If the value of the numerator for SIR is smaller than that of the denominator, factors associated with surgeons or medical institutes act to suppress SSI events. Specifically, a year with an SIR > 1 has a higher frequency of SSI events than the year 2013, indicating that the medical facility's performance was lower than that in 2013 in that year (and vice versa).

### Statistical analysis

To calculate the uncertainty bound for the crude relative risk and SIR of SSI events, we calculated the following: First, we used a Wald normal approximation interval to estimate the standard error of the relative risk, i.e.,

2 $$s.e.\left(\mathrm{ln}\left(\widehat{RR}\right)\right)=\sqrt{\frac{1}{a}-\frac{1}{a+b}+\frac{1}{c}-\frac{1}{c+d}}$$

where *a*, *b*, *c*, and *d* are the frequencies in a 2 × 2 contingency table. Regarding SIR, we used a Wald confidence interval for computing the 95% confidence intervals for SIR, i.e.,

3 $$\left(\widehat{SIR}-1.96\sqrt{\frac{\widehat{SIR}}{\sum_{\mathrm{i}=1}^{\mathrm{n}}{R}_{i}{P}_{i}}},\widehat{SIR}+1.96\sqrt{\frac{\widehat{SIR}}{\sum_{\mathrm{i}=1}^{\mathrm{n}}{R}_{i}{P}_{i}}}\right).$$

We also explored the time trends in SSI frequency, including the SSI frequency for all operations for a single surgical site and those stratified by laparoscopy, stoma construction, emergency surgery, and sex. For this purpose, we used the Cochran–Armitage trend test for a proportion over time. Statistical analyses were performed using JMP version 14 (SAS Institute Inc., Cary, NC, USA).

### Ethical concerns

We analyzed data that are publicly available from the nationwide surveillance system, JANIS [10] and the analysis of publicly available data with no identifiable information did not require ethical approval. This complies with the national guideline, “ethics guideline for life science and medical research studies involving human subjects” (https://www.meti.go.jp/press/2020/03/20210323004/20210323004-1.pdf).

## Results

**Fig. 1:**
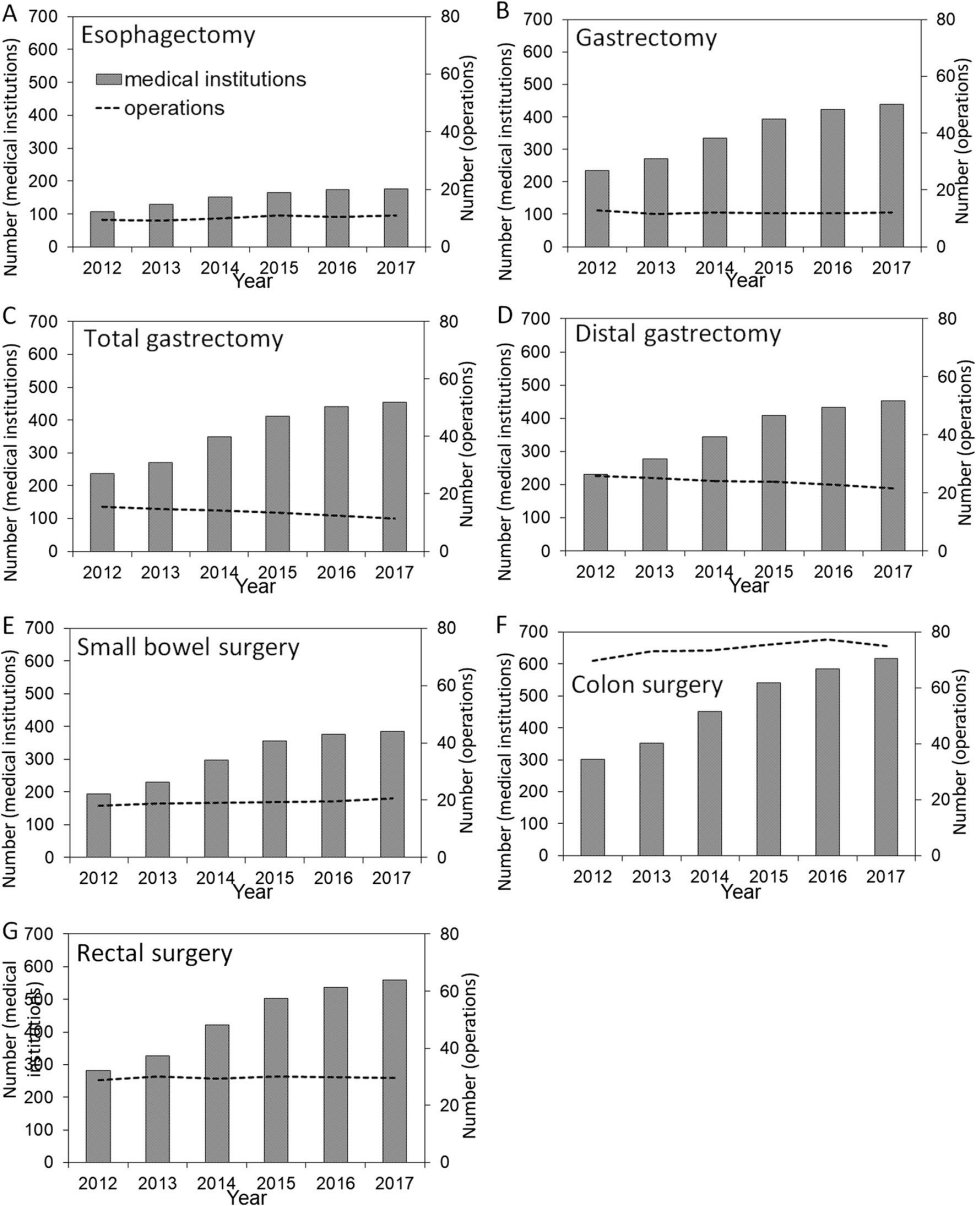
Trends in the number of medical institutions registered in JANIS and the number of surgical operations, 2012–2017. Panels are shown separately for **A** esophagectomy, **B** gastrectomy, **C** total gastrectomy, **D** distal gastrectomy, **E** small bowel surgery, **F** colon surgery, and **G** rectal surgery. Bars represent the registered number of medical institutions, and the dotted lines show the annual average number of surgical operations per registered medical institution. *JANIS* Japan Nosocomial Infections Surveillance

The number of registered medical institutes in the JANIS SSI division and the total number of operations per medical institute are shown in Fig. 1 by type of surgery. Overall, for the seven different types of gastrointestinal surgery, an increased number of registrations to the SSI division were seen from 2012 to 2017. However, trends in the number of operations per institute were not uniform; the frequency of surgery for the upper gastrointestinal tract remained nearly constant or decreased over time, while that of the lower gastrointestinal tract increased or remained unchanged.

**Fig. 2:**
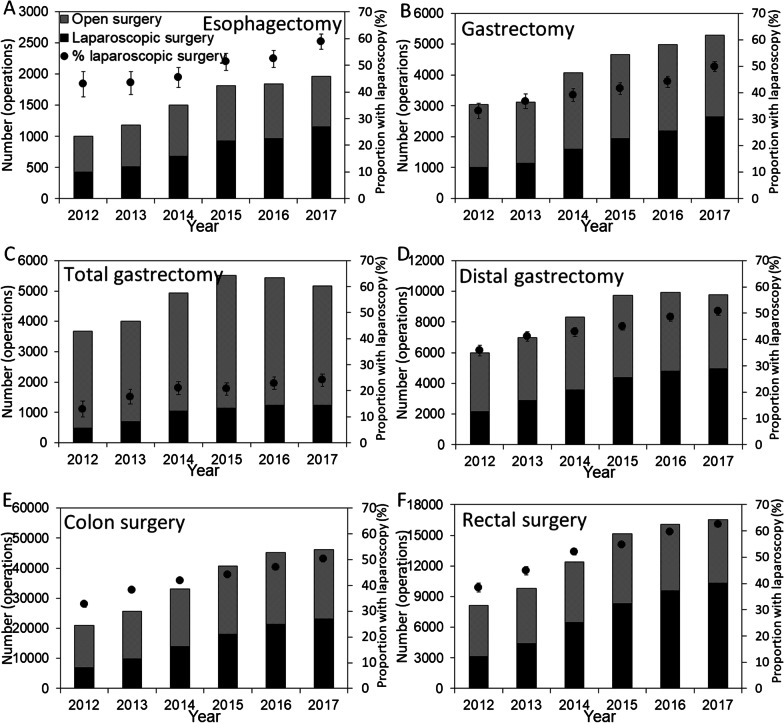
Trends in gastrointestinal surgery among the medical institutions registered in JANIS, 2012–2017. Panels are shown separately for **A** esophagectomy, **B** gastrectomy, **C** total gastrectomy, **D** distal gastrectomy, **E** colon surgery, and **F** rectal surgery. The black bars represent the number of laparoscopic surgeries, and the gray bars represent the number of open surgeries. The solid circles indicate the proportion of laparoscopic surgeries of the total number of surgical operations, and the error bars shown by whiskers indicate the 95% confidence intervals for the proportion. *JANIS* Japan Nosocomial Infections Surveillance

Figure 2 shows the number of surgical operations by the type of surgery and the use of laparoscopy over time; both the number of operations and the number of laparoscopic surgeries increased over time. For each surgical site, the trend test revealed a significant decreasing trend for open surgery (p < 0.01 for all six surgical sites other than upper gastrointestinal surgery; Table 1), indicating that the use of laparoscopic surgery increased over time.

**Table 1:** Trends in the proportion of operations by surgical technique and variable in Japan, 2012–2017

Surgical technique	Variable	Rate per year	Chi^2^	p-value
Esophagectomy	Female	0.01	0.17	0.68
	Emergency surgery	− 0.03	0.49	0.48
	Open surgery	− 0.06	111.01	< 0.01*
Gastrectomy	Female	< 0.01	0.26	0.61
	Emergency surgery	− 0.01	0.60	0.44
	Open surgery	− 0.05	283.18	< 0.01*
Total gastrectomy	Female	− 0.01	0.99	0.32
	Emergency surgery	0.07	3.12	0.08
	Open surgery	− 0.02	33.86	< 0.01*
Distal gastrectomy	Female	0.01	3.41	0.06
	Emergency surgery	0.05	4.00	0.05
	Open surgery	− 0.04	424.46	< 0.01*
Small bowel surgery	Female	< 0.01	0.63	0.43
	Emergency surgery	< 0.01	0.01	0.94
	No stoma	< 0.01	0.05	0.82
Colon surgery	Female	< 0.01	4.68	0.03*
	Emergency surgery	< 0.01	0.25	< 0.01*
	Open surgery	− 0.05	2370.33	< 0.01*
	No stoma	< 0.01	18.03	< 0.01*
Rectal surgery	Female	0.01	6.14	< 0.01*
	Emergency surgery	0.07	26.09	< 0.01*
	Open surgery	− 0.08	1789.80	< 0.01*
	No stoma	− 0.02	98.87	< 0.01*

*Chi*^*2*^ chi-square value with 1 degree of freedom

*Significant result

**Fig. 3:**
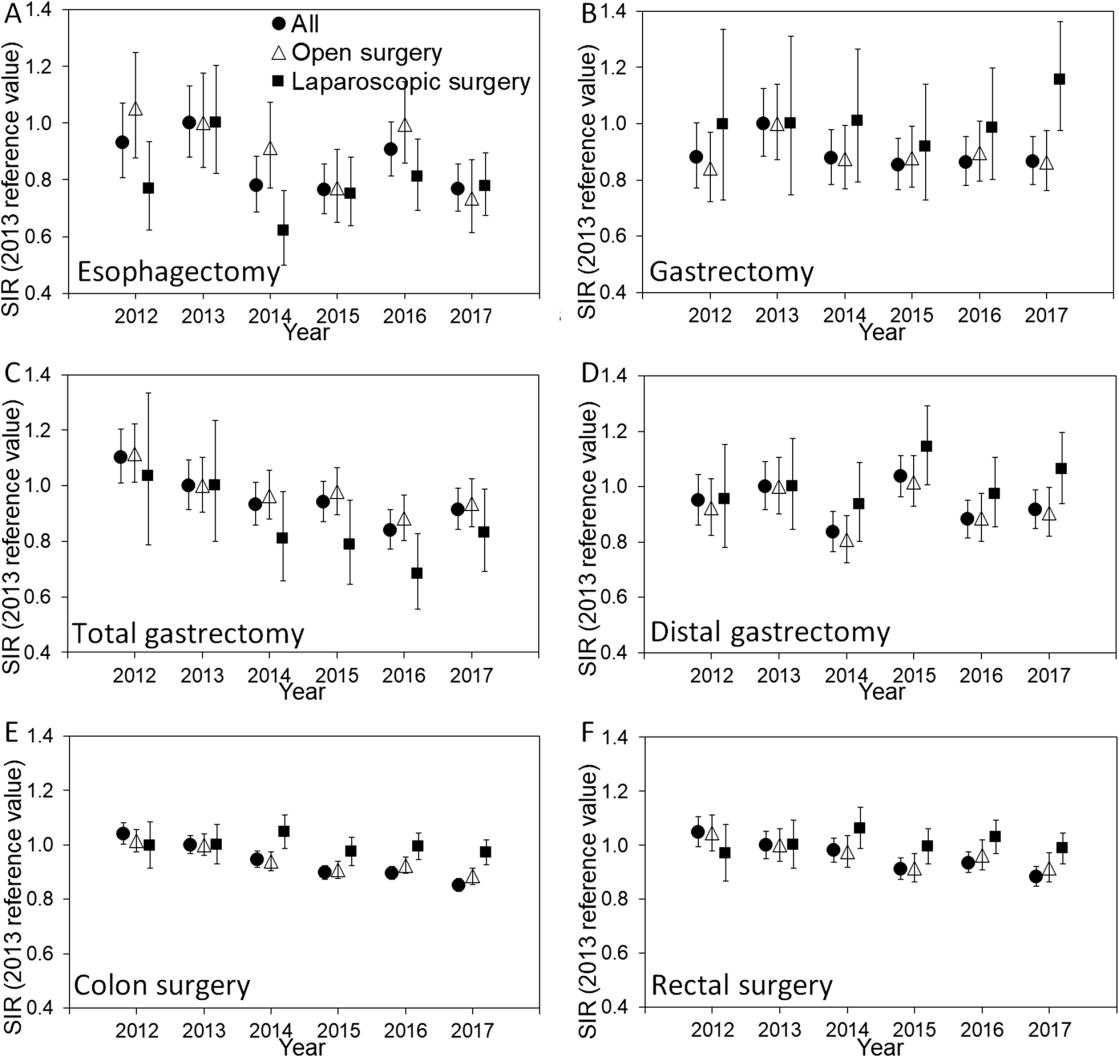
Trends in the standardized infection ratio among the medical institutions registered in JANIS, 2012–2017. Panels are shown separately for **A** esophagectomy, **B** gastrectomy, **C** total gastrectomy, **D** distal gastrectomy, **E** colon surgery, and **F** rectal surgery. The standardized infection ratio (SIR) was calculated using the 2013 data as a reference, correcting for patient status severity. A year with an SIR value > 1 had a higher frequency of surgical site infection events than in 2013, indicating that the medical facility's performance was lower than that in 2013. The solid circles represent the overall SIR numbers, and the errors bars (whiskers) indicate the 95% confidence intervals. Similarly, the open triangles and solid squares indicate SIRs for open surgery and laparoscopic surgery, respectively. *JANIS* Japan Nosocomial Infections Surveillance

**Fig. 4:**
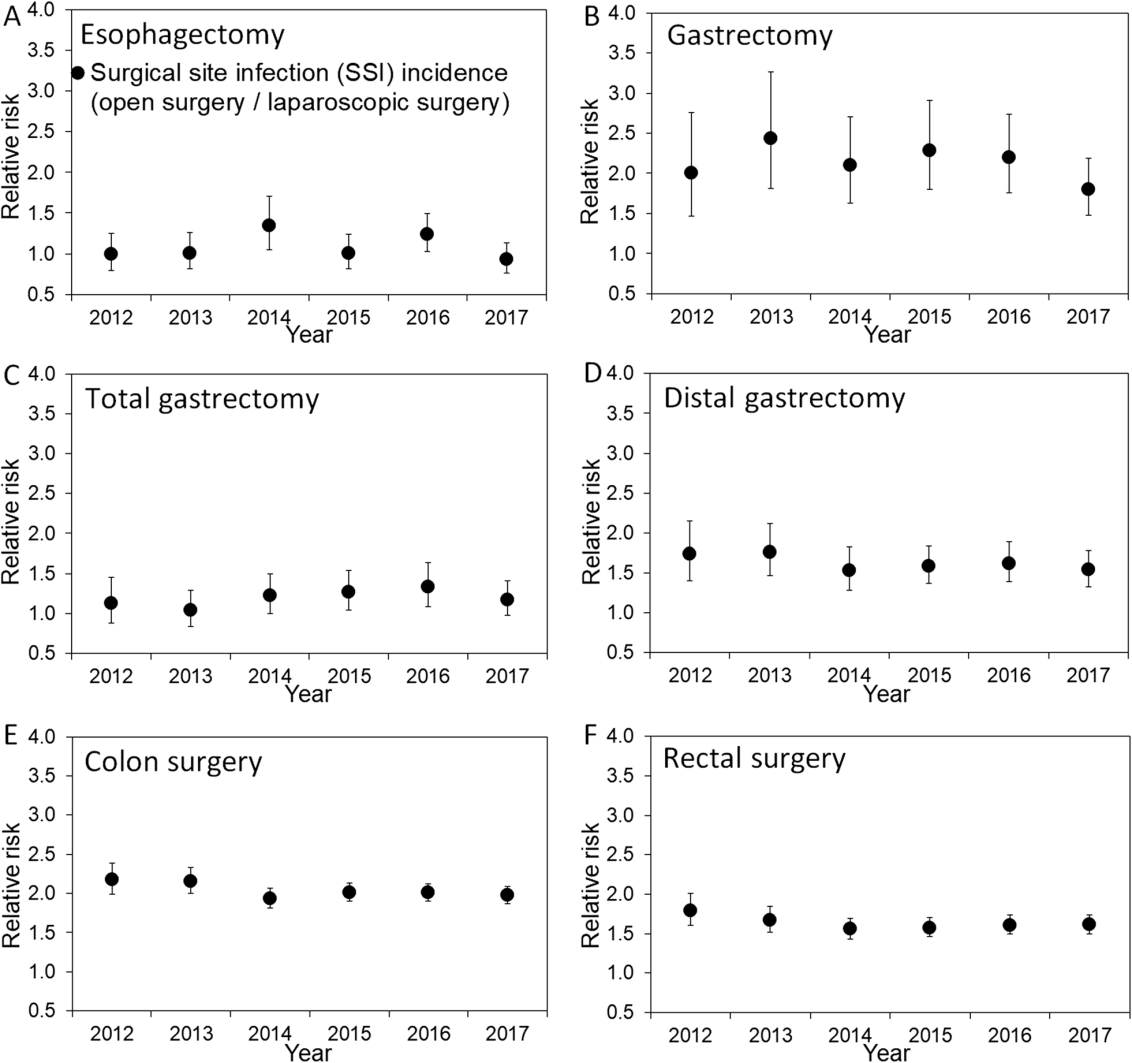
Trends in the relative risk of SSI following open vs laparoscopic surgery among the medical institutions registered in JANIS, 2012–2017. Panels are shown separately for **A** esophagectomy, **B** gastrectomy, **C** total gastrectomy, **D** distal gastrectomy, **E** colon surgery, and **F** rectal surgery. The relative risk of surgical site infections following open surgery as the exposed group (numerator) compared with that following laparoscopic surgery as the unexposed group (denominator) was calculated. The solid circles represent the estimate, and the associated error bars (whiskers) indicate the 95% confidence intervals. *SSI* surgical site infection*, JANIS* Japan Nosocomial Infections Surveillance

Time-dependent SSI risk is compared by type of surgery and the use of laparoscopy in Fig. 3. SSIs for all surgeries decreased significantly over time (Table 2). Except for gastrectomy (p = 0.37) and distal gastrectomy (p = 0.47), SSI incidence showed time-dependent decreasing trends (p = 0.01 for esophagectomy, and p < 0.01 for total gastrectomy, small bowel surgery, colon surgery, and rectal surgery; Table 2). Of the surgical sites with decreasing SIR, the frequency of SSI following laparoscopic surgery in the lower intestinal tract did not vary significantly with time (p = 0.39 for colon surgery and p = 1.00 for rectal surgery), while that for upper gastrointestinal tract surgery decreased over time (p = 0.04 for esophagectomy and p = 0.03 for total gastrectomy; Table 2). The relative risk of open surgery in causing an SSI event compared with laparoscopy is shown in Fig. 4. A relative risk > 1 was always the case for gastrectomy, distal gastrectomy, colon surgery, and rectal surgery, while this was not the case for esophagectomy and total gastrectomy, both of which involve long operation times for both open and laparoscopic/thoracoscopic surgeries. The observed SIR patterns were similar to the time-dependent trends for the crudely-calculated SSI events (Additional file 1: Figure S1).

**Table 2:** Trends in the incidence of surgical site infections in Japan, 2012–2017

Surgical technique	Variable	Rate per year	Chi^2^	p-value
Esophagectomy	All	− 0.03	6.09	0.01*
	Male	− 0.03	4.44	0.04*
	Female	0.01	10.36	< 0.01*
	Emergency surgery	− 0.09	1.50	0.22
	Elective surgery	− 0.03	2.81	0.09
	Laparoscopic surgery	− 0.03	4.08	0.04*
	Open surgery	− 0.04	6.78	0.01*
Gastrectomy	All	− 0.01	0.79	0.37
	Male	− 0.04	6.48	0.01*
	Female	0.03	1.02	0.31
	Emergency surgery	− 0.08	13.91	< 0.01*
	Elective surgery	0.02	0.82	0.36
	Laparoscopic surgery	0.04	1.56	0.21
	Open surgery	< 0.01	0.01	0.94
Total gastrectomy	All	− 0.04	16.30	< 0.01*
	Male	− 0.03	5.51	0.02*
	Female	< 0.01	0.02	0.89
	Emergency surgery	− 0.07	1.84	0.17
	Elective surgery	− 0.02	3.27	0.07
	Laparoscopic surgery	− 0.05	4.57	0.03*
	Open surgery	− 0.03	9.46	< 0.01*
Distal gastrectomy	All	− 0.01	0.52	0.47
	Male	− 0.03	4.44	0.04*
	Female	− 0.01	0.06	0.80
	Emergency surgery	− 0.08	4.66	0.03*
	Elective surgery	< 0.01	0.08	0.78
	Laparoscopic surgery	0.02	1.19	0.28
	Open surgery	< 0.01	0.06	0.81
Small bowel surgery	All	− 0.03	13.68	< 0.01*
	Male	− 0.02	3.72	0.05
	Female	− 0.03	6.53	0.01*
	Emergency surgery	− 0.03	8.99	< 0.01*
	Elective surgery	− 0.02	1.73	0.19
	Stoma	− 0.02	1.14	0.29
	No stoma	− 0.03	8.70	< 0.01*
Colon surgery	All	− 0.04	117.71	< 0.01*
	Male	− 0.03	42.18	< 0.01*
	Female	− 0.03	22.90	< 0.01*
	Emergency surgery	< 0.01	3237.22	< 0.01*
	Elective surgery	− 0.03	45.94	< 0.01*
	Laparoscopic surgery	− 0.01	0.75	0.39
	Open surgery	< 0.01	0.01	0.94
	Stoma	− 0.05	36.37	< 0.01*
	No stoma	− 0.03	39.55	< 0.01*
Rectal surgery	All	− 0.03	33.33	< 0.01*
	Male	− 0.02	10.04	< 0.01*
	Female	− 0.03	6.29	0.01*
	Emergency surgery	− 0.05	10.02	< 0.01*
	Elective surgery	− 0.02	14.48	< 0.01*
	Laparoscopic surgery	< 0.01	< 0.01	1.00
	Open surgery	− 0.02	35.80	< 0.01*
	Stoma	− 0.03	16.78	< 0.01*
	No stoma	− 0.03	12.06	< 0.01*

*Chi*^2^ chi-square value with 1 degree of freedom

*Significant result

Compared with laparoscopy, the frequency of emergency surgery and sex differences did not vary over time for surgery in the upper gastrointestinal tract, except for a marginally-significant increase in emergency surgery for distal gastrectomy (Table 1). The relative risk and SIR for each surgical type by emergency/elective surgery and stoma construction are shown in Additional file 2: Figures S2, Additional file 3: Figure S3, Additional file 4: Figure S4, Additional file 5: Figure S5. Colon and rectal surgery consistently had an RR of approximately 2.0 with no abrupt changes over time (Additional file 2: Figure S2), but the frequency of SSI following these surgeries as emergency surgeries and the sum of the number of emergency/elective surgeries decreased over time (Additional file 3: Figure S3). Regarding stoma construction, the relative risk consistently slightly exceeded 1.0 for small bowel, colon, and rectal surgery (Additional file 5: Figure S5). Significant decreases were observed for the frequency of SSIs following surgeries with concurrent stoma construction except for small bowel surgery (Additional file 5: Figure S5 & Table 2).

Time trends for the type of SSIs are shown in Additional file 6: Figure S6. The proportions of superficial and deep incisional SSIs remained stable over time.

## Discussion

The present study analyzed publicly accessible national surveillance data for nosocomial infections in Japan, focusing on temporal trends in SSI incidence. We compared the risk over time as both crude proportions and SIR, adjusting for the severity category of the SSIs. We also evaluated possible risk factors for SSI events in gastrointestinal surgery by surgical site. Overall, the frequency of SSI events, except for the upper gastrointestinal tract, revealed a clear decreasing time-dependent trend. It should be noted that the use of laparoscopic surgery increased dramatically over time, while the frequency of SSI events during laparoscopic surgery remained unchanged, implying a possibility that the increased use of laparoscopic surgery for the lower gastrointestinal tract was responsible for the decreased SSI frequency. We also evaluated emergency surgery and stoma construction regarding the overall trends in SSI events, and the frequency of SSI events during these procedures was shown to have decreased over time.

As a remarkable finding, the number of laparoscopic surgeries for all gastrointestinal surgeries increased significantly over time [21] (Fig. 2), and this was the case for the six different surgical sites, i.e., all except small bowel surgery (Table 1). In gastroenterology, laparoscopic surgery requires surgical skill but is also associated with less bleeding, shorter hospital stay, and faster recovery of gastrointestinal function, compared with open surgery [17, 22–26]. Except for small bowel surgery, which is frequently performed as an emergency operation, SSI incidence decreased for four surgical procedures, namely, esophagectomy, total gastrectomy, colon surgery, and rectal surgery. In particular, the SSI incidence for colon and rectal surgery decreased significantly when stratified by sex, emergency/elective surgery, and with/without stoma construction, while the trend for laparoscopic surgery remained unchanged over time. These findings imply that the increasing use of laparoscopic surgery had a potential causal impact on the decreasing SSI risk, especially in lower gastrointestinal tract surgery. Although the present study is observational study of aggregated data and thus, cannot conclude the causality, the finding is partly explainable by SSI countermeasures for lower gastrointestinal surgery, namely, preoperative mechanical preparation and preoperative oral antibiotic prophylaxis, which have been recommended with high-level scientific evidence [27]. In fact, a significant decreasing trend for SIR following open colon surgery is consistent with improved preoperative management over time (Fig. 3E). However, preoperative management and postoperative antibiotics cannot fully explain our findings; i.e., the relative risk of SSI remained stable over time (Fig. 4), and the addition of antibiotic prophylaxis recommended in the 2016 guidelines did not abruptly change the trend in SSI (Fig. 3). Laparoscopic surgery was an important factor in reducing the risk of SSI in a published study from Japan [13, 28]. Considering laparoscopic survey involves faster recovery and wound healing, shorter length of stay, and reduced frequency infections compared with open surgery, biological plausibility of causal link is well supported. If the causal link is real, the overall SSI incidence among previously healthy individuals is expected to decrease, because the use of laparoscopic surgery has high growth potential in Japan. In future studies, to analyze the data to interpret the SSI trends, it will be important to distinguish the SSI event frequency between laparoscopic and open surgery.

Variables other than laparoscopy revealed characteristic results. SSI events decreased significantly following emergency surgery in the upper gastrointestinal tract, especially during gastrectomy and small bowel surgery, while SSI events associated with elective surgery remained unchanged. Moreover, the risk of SSI events among men undergoing gastrectomy, total gastrectomy, and distal gastrectomy decreased over time, while those among women did not decrease significantly. The exact reasons for these observations must be clarified, but improved SSI control in gastrectomy during emergency surgery and recognizing high-risk patients preoperatively could have resulted in these differences. The SSI risk associated with small bowel surgery decreased significantly with stoma construction, while that associated with surgery without stoma construction did not change over time. Again, improved SSI control during stoma construction could have contributed to the decrease.

To our knowledge, the present study is the first descriptive analysis of SSI events for gastrointestinal surgery in Japan, explicitly analyzing the time trends and stratifying the SSI risk by multiple variables. Only with this type of analysis, can we systematically improve data collection (e.g., stratification by laparoscopic and open surgery) and potentially evaluate perisurgical infection control practices (e.g., improved SSI control in gastrectomy during emergency surgery). Moreover, we anticipate decreasing future trends in SSI incidence, given an increasing trend in the use of laparoscopic surgery and expected improved early detection of malignant neoplasms in the colon and rectum. However, patients undergoing open surgery of the lower gastrointestinal tract are particularly at risk of SSIs. This issue is not restricted to patients’ background characteristics, which were adjusted during the SIR calculations. What was adjusted during the calculation of SIR includes (i) the length of operation, (ii) severity of patients, and (iii) classification of wound (i.e., extent of bacterial contamination), and in fact, other important variables including transfusion during the operation, the drug use of diabetic patients and ages were not addressed [13]. Among diabetic patients undergoing elective surgery, laparoscopic surgery is performed after improving blood glucose concentrations, which is a known risk factor for SSI [13]. However, in emergency surgery, the blood glucose concentration cannot be adjusted preoperatively, and as a result, open surgery, which is the only option in emergency events, must involve the risk of SSI. Moreover, oral antibiotics cannot be administered sufficiently in advance of emergency surgery. For these reasons, selection bias and confounders inherently influence the causal interpretation of the SSI rate in relation to the increasing proportion of laparoscopic surgery. Considering that indications for an open surgery are different from those for a laparoscopic approach, patients who require open surgery may possess greater number of preexisting factors (e.g. more advanced disease) or involve greater complexity of surgical operation compared with laparoscopic group. It is extremely hard to address these issues using database, and thus, these points were not within the scope of our study.

The present study was not free from limitations. First, we evaluated aggregated surveillance data, and thus, the risk was not calculable per medical institute. Second, for the same reason, the use of secondary data did not permit us to control numerous confounders. Two other studies from Japan [13, 14] analyzed individual-based epidemiological datasets and better identified SSI risk factors compared with our study. Third, because of the nature of surveillance data, we were not able to stratify SSI events by the time from the surgery or by causative agents. Fourth, because we focused on time-dependent trends in SSI events regarding the accessible number of variables, other underlying risk factors for SSI; e.g., age and underlying comorbidities such as diabetes mellitus, use of steroids, and intraoperative blood transfusion [13], were not controlled.

Despite the limitations associated with the nature of surveillance data, we believe that the present study is unique in that it allowed an objective interpretation of the time-dependent trends in SSI event risks statistically associated with gastrointestinal surgery in Japan. Our results showed a stable lower risk of SSI following laparoscopic surgery over time, as well as implying that the increased use of laparoscopic surgery may explain the time-dependent decline in SSI events over time, in Japan.

## Conclusions

The use of laparoscopic surgery increased significantly over the study period, except for surgery in the small bowel. The increased proportion of laparoscopic surgery may have had an impact on the decrease in SSI risk seen in our study, especially for lower gastrointestinal tract surgery. If the causal link is real, the overall SSI incidence among previously healthy individuals is expected to decrease, because laparoscopic surgery has large growth potential in Japan.

## Supplementary Information

**Additional file 1: Figure S1. **Trends in the incidence of surgical site infections among the medical institutions registered in JANIS, 2012–**2017.** Panels are shown separately for **A** esophagectomy, **B** gastrectomy, **C** total gastrectomy, **D** distal gastrectomy, **E** colon surgery, and **F** rectal surgery. The solid circles indicate the overall surgical site infection (SSI) incidence rates, with error bars shown by whiskers indicating the 95% confidence intervals. Open triangles and solid squares show the incidence of SSI following open surgery and laparoscopic surgery, respectively. *JANIS* Japan Nosocomial Infections Surveillance

**Additional file 2: Figure S2.** Trends in the relative risk of surgical site infections following emergency operation compared with elective surgery among the medical institutions registered in JANIS, 2012–2017. Panels are shown separately for **A** esophagectomy, **B** gastrectomy, **C** total gastrectomy, **D** distal gastrectomy, **E** small bowel surgery, **F** colon surgery, and **G** rectal surgery. The relative risk of surgical site infections following emergency operation as the exposed group (numerator) compared with that following elective surgery as the unexposed group (denominator) was calculated. The solid circles represent the estimate, with their error bars shown by whiskers indicating the 95% confidence intervals. *JANIS* Japan Nosocomial Infections Surveillance

**Additional file 3:** **Figure S3.** Trends in the incidence of surgical site infections by the type of surgery among the medical institutions registered in JANIS, 2012–2017. Panels are separately shown for **A** esophagectomy, **B** gastrectomy, **C** total gastrectomy,** D** distal gastrectomy, E small bowel surgery, **F** colon surgery, and **G** rectal surgery. The solid circles indicate the proportion of surgical site infections (SSI) of all surgical operations, with error bars shown by whiskers indicating the 95% confidence intervals. The open triangles and solid squares show the incidence of SSI following emergency and elective surgery, respectively. JANIS Japan Nosocomial Infections Surveillance.

**Additional file 4: Figure S4. **Trends in the relative risk of surgical site infections comparing with and without stoma construction among the medical institutions registered in JANIS, 2012–2017. Panels are shown separately for **A** small bowel surgery, **B** colon surgery, and **C** rectal surgery. The relative risk of surgical site infections with stoma construction as the exposed group (numerator) compared with that without stoma construction as the unexposed group (denominator) was calculated. The solid circles represent the estimate, with their error bars shown by whiskers indicating the 95% confidence intervals. *JANIS* Japan Nosocomial Infections Surveillance

**Additional file 5: Figure S5.** Trends in the incidence of surgical site infections by the presence of a stoma among the medical institutions registered in JANIS, 2012–2017. Panels are shown separately for **A** small bowel surgery, **B** colon surgery, and **C** rectal surgery. The solid circles indicate the proportion of surgical site infections (SSI) among all surgical operations, with error bars shown by whiskers indicating the 95% confidence intervals. The open triangles and solid squares show the incidence of SSI with and without stoma construction, respectively. *JANIS* Japan Nosocomial Infections Surveillance

**Additional file 6: Figure S6.** Trends in the type of surgical site infections among the medical institutions registered in JANIS, 2012–2017. Of the total of all surgical site infections (SSI), percentages by the type of infections (i.e., superficial incisional, deep incisional, or organ/space infections) are given. *JANIS* Japan Nosocomial Infections Surveillance

## Data Availability

Collected datasets are publicly available and can be retrieved from the Japan Nosocomial Infections Surveillance, Ministry of Health, Labour and Welfare [10], https://janis.mhlw.go.jp/report/ssi.html.

## Abbreviations

SSI: Surgical site infection
JANIS: Japan Nosocomial Infections Surveillance
SIR: Standardized infection ratio

## References

[1] Magill SS, Edwards JR, Bamberg W, Beldavs ZG, Dumyati G, Kainer MA (2014). Emerging infections program healthcare-associated infections and antimicrobial use prevalence survey team. Multistate point-prevalence survey of health care-associated infections. N Engl J Med.

[2] Klevens RM, Edwards JR, Richards CL, Horan TC, Gaynes RP, Pollock DA (2007). Estimating healthcare-associated infections and deaths in U.S. Hospitals, 2002. Public Health Rep.

[3] Mu Y, Edwards JR, Horan TC, Berrios-Torres SI, Fridkin SK (2011). Improving risk-adjusted measures of surgical site infection for the National Healthcare Safety Network. Infect Control Hosp Epidemiol.

[4] Scott II RD. The direct medical costs of healthcare-associated infections in U.S. hospitals and the benefits of prevention. 2009. http://www.cdc.gov/ncidod/dhqp/pdf/Scott_CostPaper.pdf. Accessed 2 Jun 2019.

[5] Astagneau P, Rioux C, Golliot F, Brücker G, INCISO Network Study Group (2001). Morbidity and mortality associated with surgical site infections: results from the 1997–1999 INCISO surveillance. J Hosp Infect.

[6] de Lissovoy G, Fraeman K, Hutchins V, Murphy D, Song D, Vaughn BB (2009). Surgical site infection: incidence and impact on hospital utilization and treatment costs. Am J Infect Control.

[7] Eagye KJ, Nicolau DP (2009). Deep and organ/space infections in patients undergoing elective colorectal surgery: incidence and impact on hospital length of stay and costs. Am J Surg.

[8] Badia JM, Casey AL, Petrosillo N, Hudson PM, Mitchell SA, Crosby C (2018). Impact of surgical site infection on healthcare costs and patient outcomes: a systematic review in six European countries. J Infect Control.

[9] Saeed MJ, Dubberke ER, Fraser VJ, Olsen MA (2015). Procedure-specific surgical site infection incidence varies widely within certain National Healthcare Safety Network surgery groups. Am J Infect Control.

[10] Ministry of Health, Labour and Welfare, Japan. Japan Nosocomial Infections Surveillance. Surgical Site Infection Section. Tokyo: Ministry of Health, Labour and Welfare, 2017. https://janis.mhlw.go.jp/report/ssi.html.2018/6/18/SSI_Open_Report_201700.xlsm. Accessed 19 Oct 2021.

[11] Chida K, Watanabe J, Suwa Y, Suwa H, Momiyama M, Ishibe A (2019). Risk factors for incisional surgical site infection after elective laparoscopic colorectal surgery. Ann Gastroenterol Surg.

[12] Kohut AY, Liu JJ, Stein DE, Sensenig R, Poggio JL (2015). Patient-specific risk factors are predictive for postoperative adverse events in colorectal surgery: an American College of Surgeons National Surgical Quality Improvement Program-based analysis. Am J Surg.

[13] Schilling PL, Dimick JB, Birkmeyer JD (2008). Prioritizing quality improvement in general surgery. J Am Coll Surg.

[14] Fukuda H (2016). Patient-related risk factors for surgical site infection following eight types of gastrointestinal surgery. J Hosp Infect.

[15] Morikane K, Honda H, Yamagishi T, Suzuki S, Aminaka M (2014). Factors associated with surgical site infection in colorectal surgery: the Japan nosocomial infections surveillance. Infect Control Hosp Epidemiol.

[16] Astagneau P, Aupee M, Behnke M, Bull A, Choi HJ, de Greeff SC (2018). Impact of participation in a surgical site infection surveillance network: results from a large international cohort study. J Hosp Infect.

[17] Choi HJ, Adiyani L, Sung J, Choi JY, Kim HB, Kim YK, Korean Nosocomial Infections Surveillance System (KONIS) (2016). Five-year decreased incidence of surgical site infections following gastrectomy and prosthetic joint replacement surgery through active surveillance by the Korean Nosocomial Infection Surveillance System. J Hosp Infect.

[18] Committee for the Guidelines for Prevention of Surgical Site Infections (2018). Guidelines for perioperative management for the prevention of surgical site infections following gastrointestinal surgery, 2018.

[19] Sakamoto F (2013). Studying interventions against healthcare-associated infections: from standard precautions to surveillance.

[20] Japan Nosocomial Infection Surveillance Network (JANIS). https://janis.mhlw.go.jp/english/index.asp. Accessed: 5 Dec 2019

[21] Fukuda H (2014). Evaluation and analysis of surgical site infection surveillance data. J Jpn Soc Infect Prev Control.

[22] Ma Y, Yang Z, Qin H, Wang Y (2011). A meta-analysis of laparoscopy compared with open colorectal resection for colorectal cancer. Med Oncol.

[23] Nishidate T, Furuhata T, Okita K, Ueki T, Akizuki E, Meguro M (2014). Risk factors of surgical site infection (SSI) in rectal surgery. J Jpn Soc Surg Infect.

[24] Yoshida K, Honda M, Kumamaru H, Kodera Y, Kakeji Y, Hiki N (2018). Surgical outcomes of laparoscopic distal gastrectomy compared to open distal gastrectomy: a retrospective cohort study based on a nationwide registry database in Japan. Ann Gastroenterol Surg.

[25] Chen XZ, Wen L, Rui YY, Liu CX, Zhao QC, Zhou ZG (2015). Long-term survival outcomes of laparoscopic versus open gastrectomy for gastric cancer: a systematic review and meta-analysis. Medicine.

[26] Schnitzbauer V, Gerken M, Benz S, Völkel V, Draeger T, Fürst A (2019). Laparoscopic and open surgery in rectal cancer patients in Germany: short and long-term results of a large 10-year population-based cohort. Surg Endosc.

[27] Davis CH, Gaglani T, Moore LW, Du XL, Hwang H, Yamal JM (2019). Trends and outcomes in laparoscopic versus open surgery for rectal cancer from 2005 to 2016 using the ACS-NSQIP database, a retrospective cohort study. Int J Surg.

[28] Japan Society for Surgical Infection. Guidelines for the appropriate use of preoperative antimicrobial prophylaxis. Japanese Society of Chemotherapy/ Japan Society for Surgical Infection. Edited by the Guidelines Committee on Appropriate Use of Antimicrobial Agents for Postoperative Infection Prevention. J Jpn Soc Surg Infect 2016;13:79–158 in Japanese

[29] Sawa A, Morikane K, Harihara Y, Shimizu J (2018). Report on JHAIS Committee and SSI Surveillance (No 19). J Jpn Soc Infect Prev Control.

