# Supplementary figures and images for: Increasing rates of laparoscopic gastrointestinal surgery and decreasing rates of surgical site infections: an observational study in Japan from 2012–2017
Source: BMC Surg. 2021 Oct 20;21:370. doi: 10.1186/s12893-021-01373-2 (PMC8527652; doi:10.1186/s12893-021-01373-2)

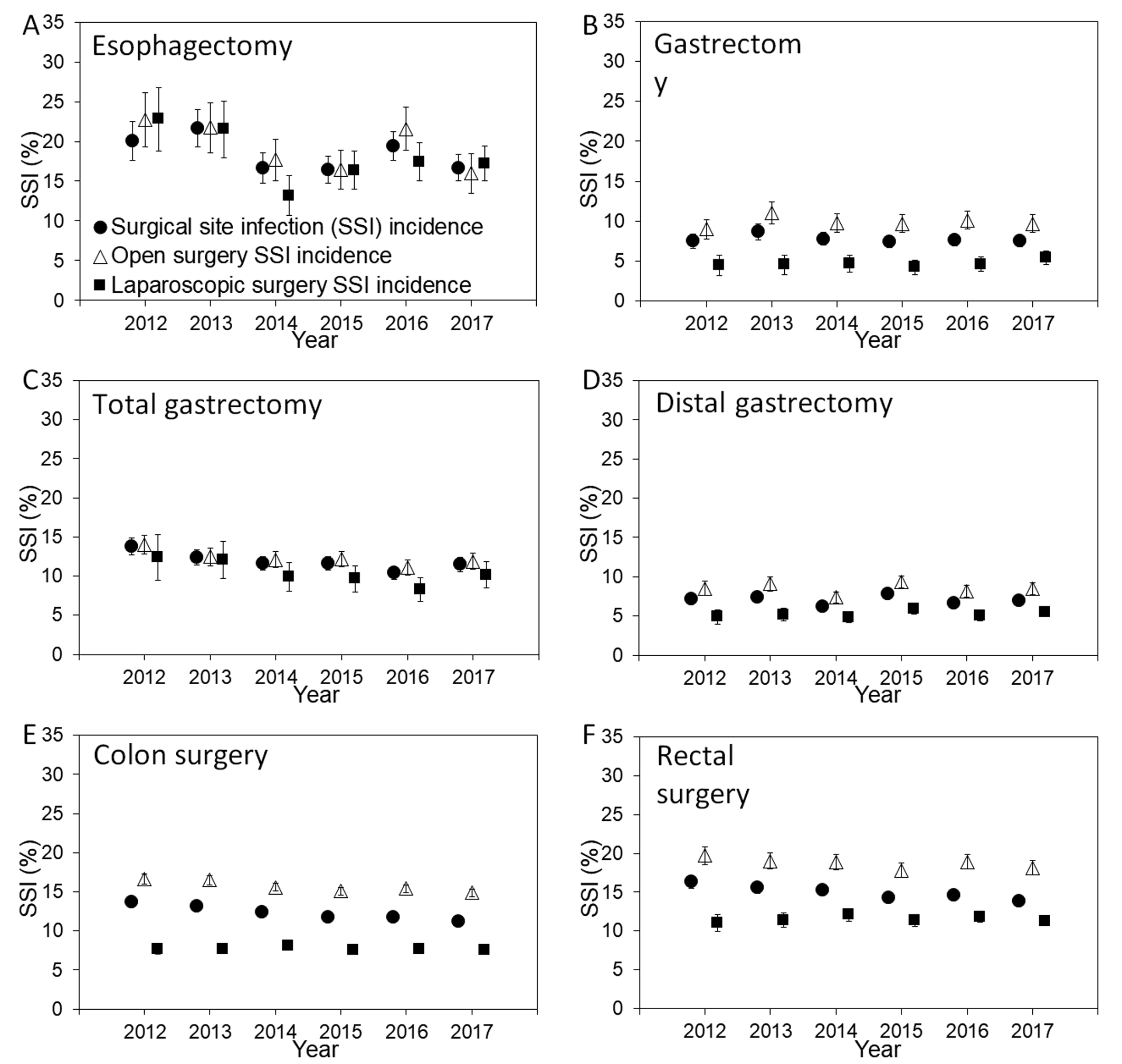

Supplement: Supplementary file 1 — Additional file 1: Figure S1. Trends in the incidence of surgical site infections among the medical institutions registered in JANIS, 2012–2017. Panels are shown separately for A esophagectomy, B gastrectomy, C total gastrectomy, D distal gastrectomy, E colon surgery, and F rectal surgery. The solid circles indicate the overall surgical site infection (SSI) incidence rates, with error bars shown by whiskers indicating the 95% confidence intervals. Open triangles and solid squares show the incidence of SSI following open surgery and laparoscopic surgery, respectively. JANIS Japan Nosocomial Infections Surveillance [file 12893_2021_1373_MOESM1_ESM.tif]

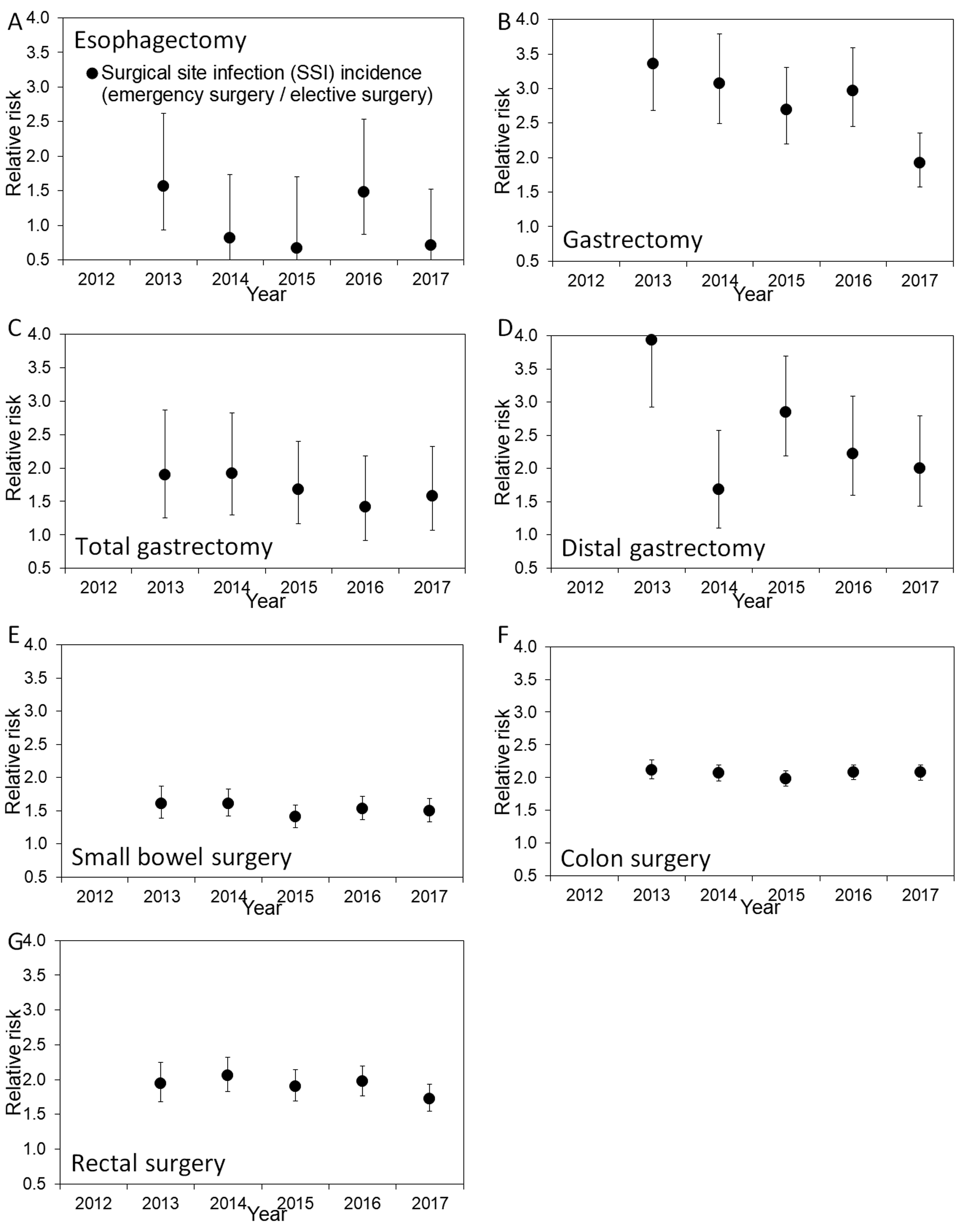

Supplement: Supplementary file 2 — Additional file 2: Figure S2. Trends in the relative risk of surgical site infections following emergency operation compared with elective surgery among the medical institutions registered in JANIS, 2012–2017. Panels are shown separately for A esophagectomy, B gastrectomy, C total gastrectomy, D distal gastrectomy, E small bowel surgery, F colon surgery, and G rectal surgery. The relative risk of surgical site infections following emergency operation as the exposed group (numerator) compared with that following elective surgery as the unexposed group (denominator) was calculated. The solid circles represent the estimate, with their error bars shown by whiskers indicating the 95% confidence intervals. JANIS Japan Nosocomial Infections Surveillance [file 12893_2021_1373_MOESM2_ESM.tif]

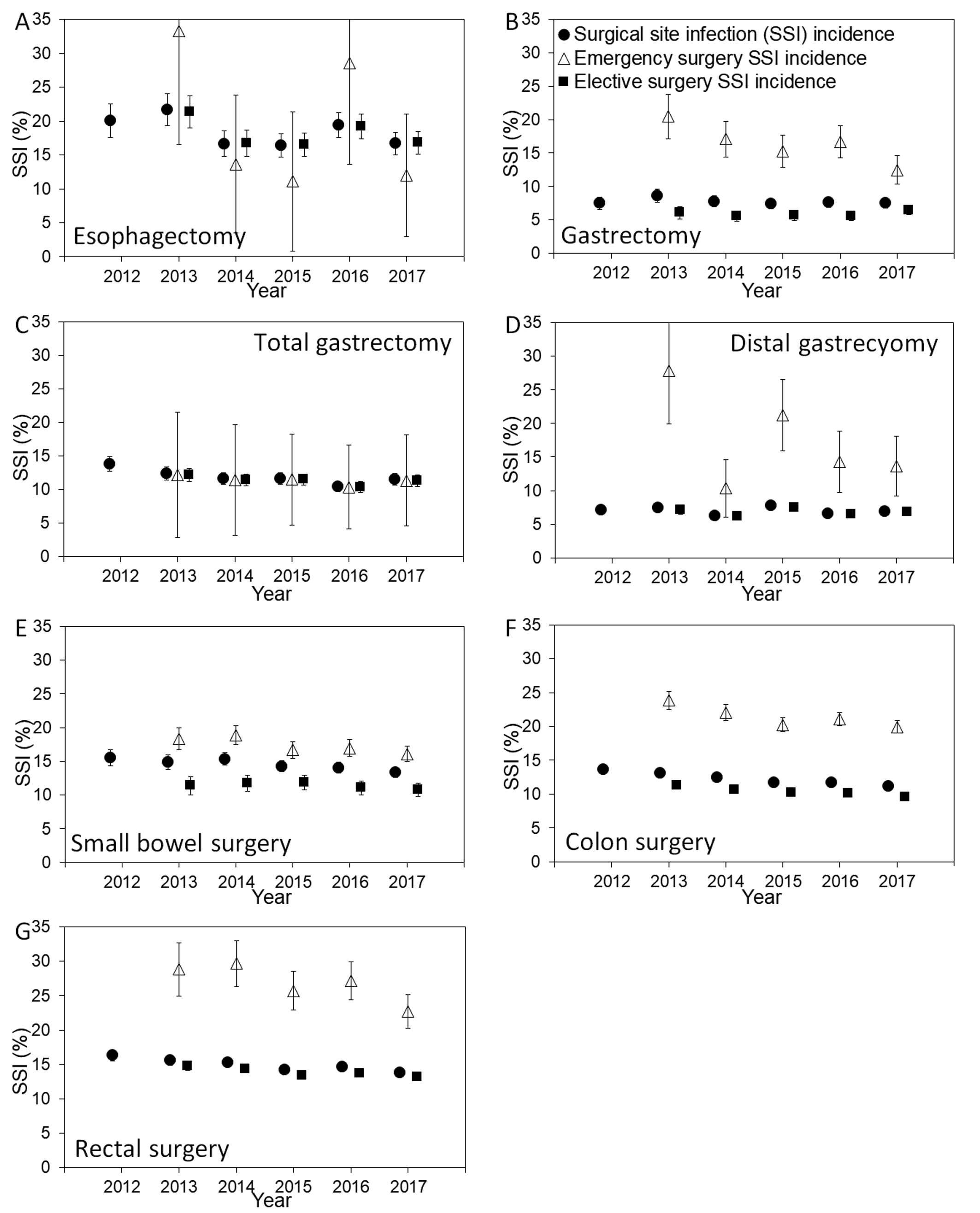

Supplement: Supplementary file 3 — Additional file 3: Figure S3. Trends in the incidence of surgical site infections by the type of surgery among the medical institutions registered in JANIS, 2012–2017. Panels are separately shown for A esophagectomy, B gastrectomy, C total gastrectomy, D distal gastrectomy, E small bowel surgery, F colon surgery, and G rectal surgery. The solid circles indicate the proportion of surgical site infections (SSI) of all surgical operations, with error bars shown by whiskers indicating the 95% confidence intervals. The open triangles and solid squares show the incidence of SSI following emergency and elective surgery, respectively. JANIS Japan Nosocomial Infections Surveillance. [file 12893_2021_1373_MOESM3_ESM.tif]

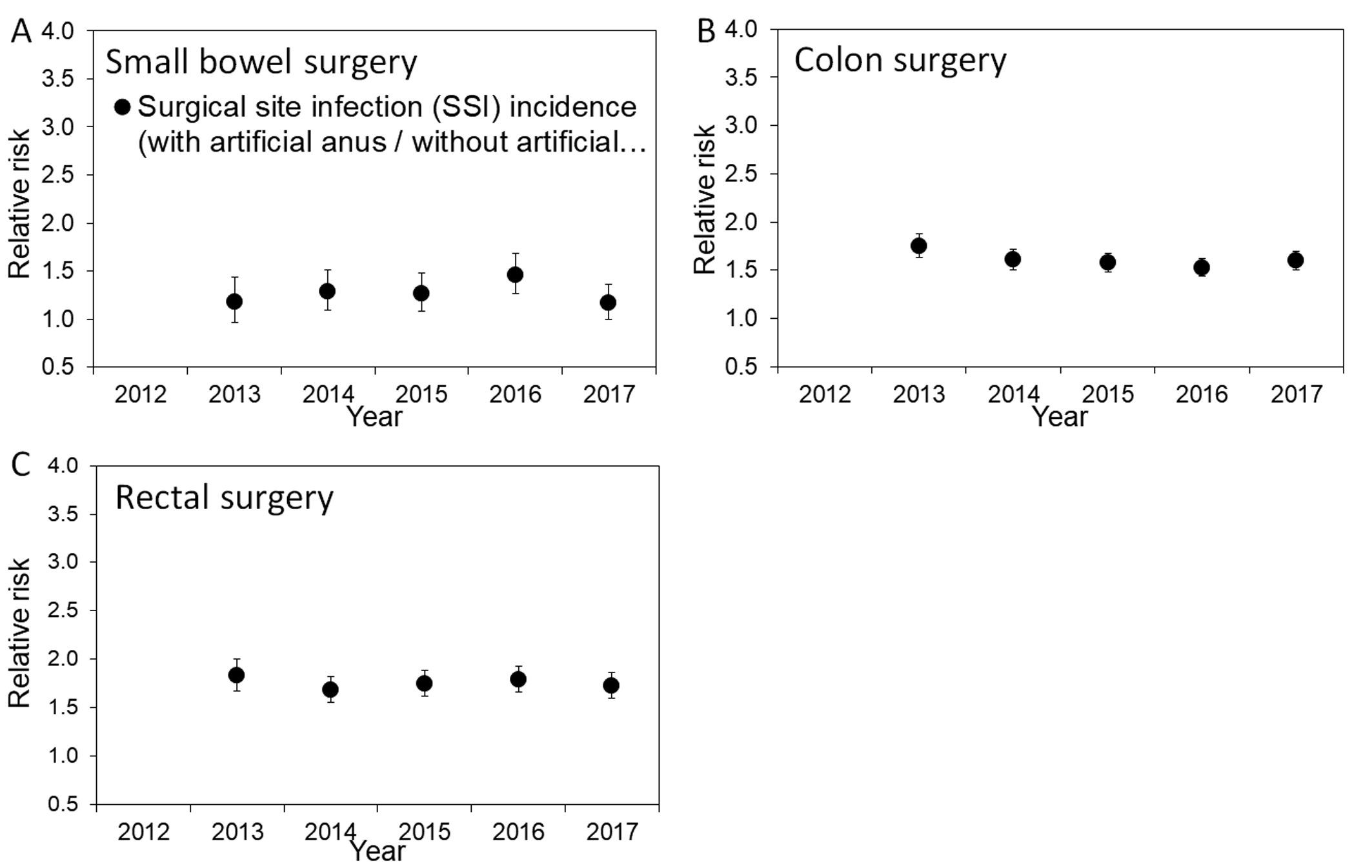

Supplement: Supplementary file 4 — Additional file 4: Figure S4. Trends in the relative risk of surgical site infections comparing with and without stoma construction among the medical institutions registered in JANIS, 2012–2017. Panels are shown separately for A small bowel surgery, B colon surgery, and C rectal surgery. The relative risk of surgical site infections with stoma construction as the exposed group (numerator) compared with that without stoma construction as the unexposed group (denominator) was calculated. The solid circles represent the estimate, with their error bars shown by whiskers indicating the 95% confidence intervals. JANIS Japan Nosocomial Infections Surveillance [file 12893_2021_1373_MOESM4_ESM.tif]

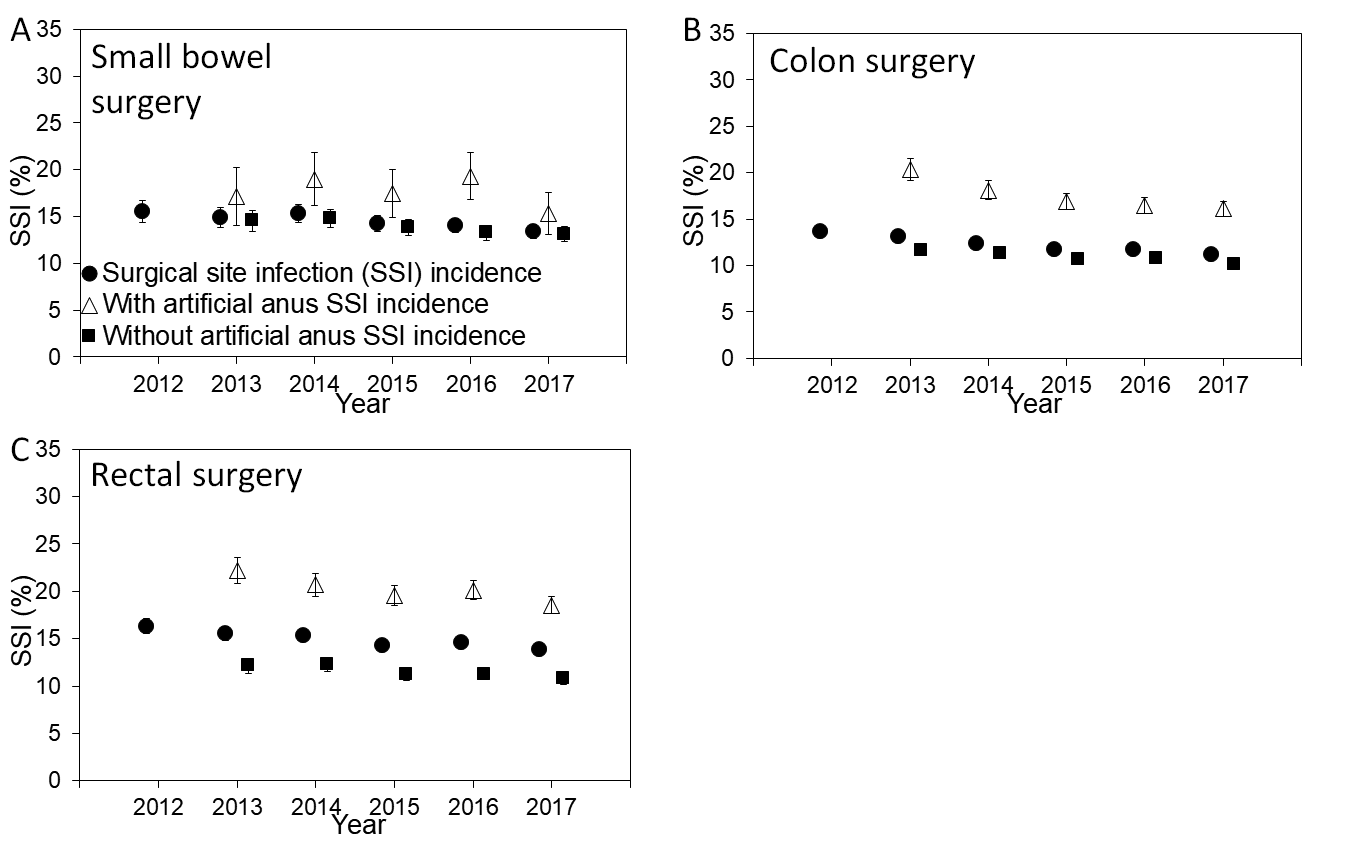

Supplement: Supplementary file 5 — Additional file 5: Figure S5. Trends in the incidence of surgical site infections by the presence of a stoma among the medical institutions registered in JANIS, 2012–2017. Panels are shown separately for A small bowel surgery, B colon surgery, and C rectal surgery. The solid circles indicate the proportion of surgical site infections (SSI) among all surgical operations, with error bars shown by whiskers indicating the 95% confidence intervals. The open triangles and solid squares show the incidence of SSI with and without stoma construction, respectively. JANIS Japan Nosocomial Infections Surveillance [file 12893_2021_1373_MOESM5_ESM.tif]

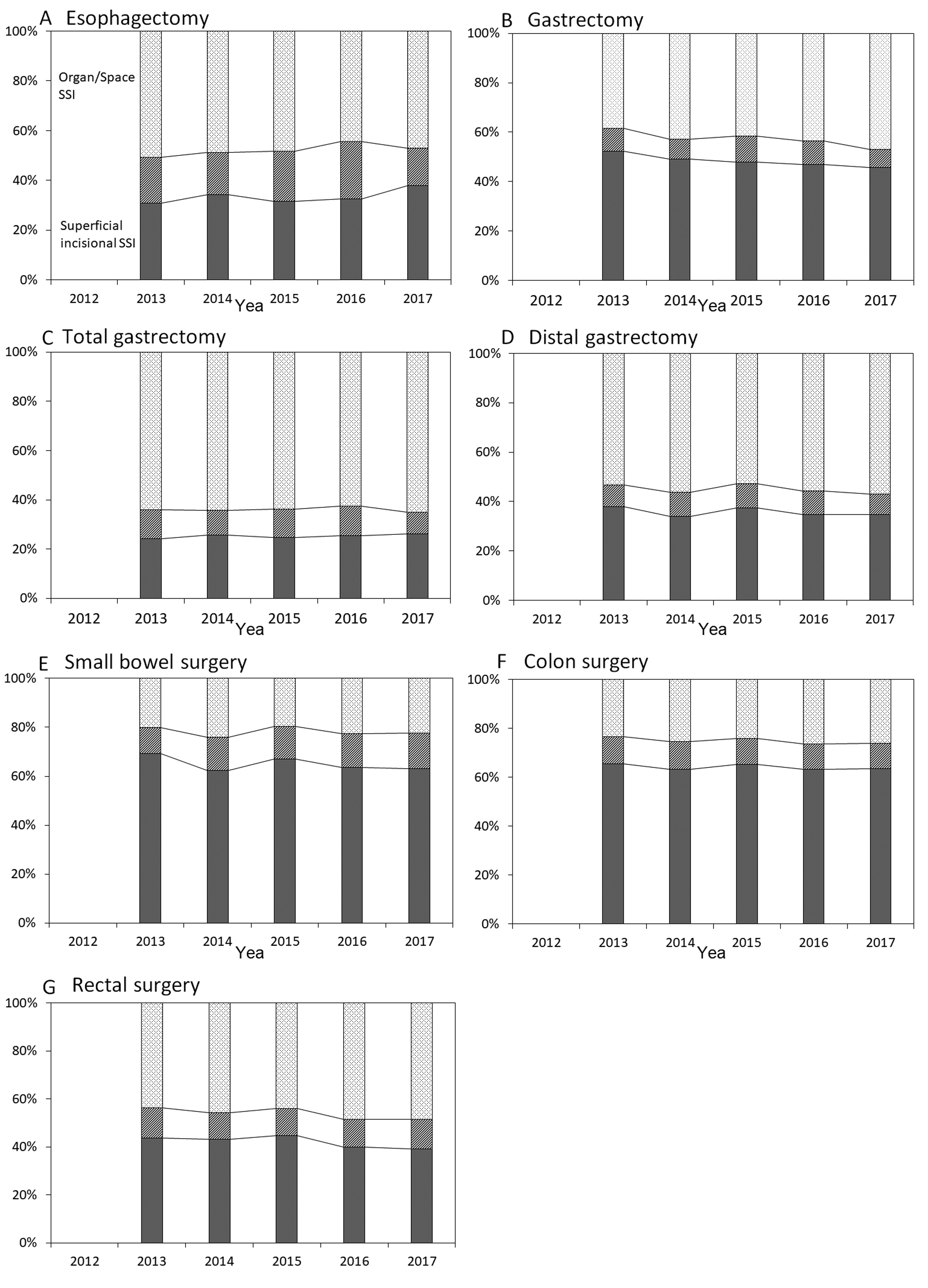

Supplement: Supplementary file 6 — Additional file 6: Figure S6. Trends in the type of surgical site infections among the medical institutions registered in JANIS, 2012–2017. Of the total of all surgical site infections (SSI), percentages by the type of infections (i.e., superficial incisional, deep incisional, or organ/space infections) are given. JANIS Japan Nosocomial Infections Surveillance [file 12893_2021_1373_MOESM6_ESM.tif]
